# Motor Fiber Tract Changes Following iTBS Stimulation in a Basal Ganglia Stroke Patient: A Case Report

**DOI:** 10.1002/ccr3.71728

**Published:** 2026-01-02

**Authors:** Lei Xi, Qian Liu, Yuzhe Zou, Xue Yang, Liqing Yao

**Affiliations:** ^1^ Department of Rehabilitation Medicine The Second Affiliated Hospital of Kunming Medical University Kunming China; ^2^ Department of Rehabilitation Medicine The Second Affiliated Hospital of Kunming Medical University/Rehabilitation College of Kunming Medical University Kunming China

**Keywords:** case report, fiber tract, iTBS, stroke

## Abstract

This case report describes the application of intermittent theta‐burst stimulation (iTBS) targeting the cerebellum in a 46‐year‐old female patient with basal ganglia infarction. Following a 2‐week intervention, enhanced corticospinal tract connectivity and contralateral compensatory fiber reorganization in specific brain regions were observed. These findings suggest that cerebellar iTBS may promote neuroplastic reorganization after basal ganglia stroke, underscoring the important role of the cerebello‐cortical pathway in functional recovery.

## Summary

Intermittent theta‐burst stimulation applied to the cerebellum can promote enhanced corticospinal tract connectivity and contralateral compensatory fiber reorganization in patients with basal ganglia stroke, thereby improving their motor, cognitive, and emotional functions.

## 1 Introduction

Motor dysfunction after stroke is a common and serious sequela, often caused by damage to cortical and subcortical motor pathways. Restoring these impaired fiber tracts is considered essential for functional recovery [1]. Transcranial magnetic stimulation (TMS), particularly the iTBS protocol, has been demonstrated as a non‐invasive technique capable of modulating cortical excitability and promoting neuroplasticity [2]. Although the cerebellum is increasingly recognized as a promising TMS target for improving balance, coordination, and mood in stroke patients, direct evidence regarding its effects on the structural integrity of specific motor fiber tracts remains limited. Importantly, the anatomical and functional basis of this intervention, especially the cerebellar‐thalamic‐basal ganglia pathway, can be visualized and quantified using diffusion tensor imaging (DTI). This case report examines structural changes in motor fiber tracts following cerebellar iTBS therapy in a patient with basal ganglia infarction using DTI, underscoring its potential to induce remote neuroplastic reorganization.

## 2 Case Presentation

On February 1, 2022, a 46‐year‐old woman suddenly experienced dizziness during sleep and initially did not pay much attention to it. By 14:00 the next day, she developed weakness in her right limb, slurred speech, and difficulty swallowing, prompting her to seek treatment at another hospital. A headComputed Tomography (CT) scan revealed an infarction in the left basal ganglia. She has a history of hypertension (controlled with amlodipine besylate) and a previous right knee fracture. Her medical history also includes surgery on her right knee and a cesarean section.

### 2.1 Differential Diagnosis, Investigations, and Treatment

On February 16, 2022, the patient was transferred to our department, still presenting with residual right‐sided hemiplegia, speech disorders, poor appetite, and insomnia. Neurological examination revealed: flattening of the right nasolabial fold, slight deviation of the mouth angle to the left, reduced superficial sensation on the right side, and paralysis of the right limb. Based on Magnetic resonance imaging (MRI) findings, a final diagnosis of ischemic stroke in the left basal ganglia (Trial of Org 10,172 in Acute Stroke Treatment, TOAST classification: large‐artery atherosclerosis) was confirmed.

Following hospitalization, the patient underwent a two‐week course of iTBS (a specialized form of TMS). The stimulation target was localized to the contralateral cerebellar hemisphere (1 cm lateral to the inion). Treatment parameters included daily 6‐min sessions, with intensity set to 80% of the active motor threshold, delivering 1200 pulses per session. A figure‐of‐eight coil (model B9076, 92 mm diameter; TMS device NS5000; Yirui De Co., Wuhan, China) was used, positioned tangentially to the scalp with the handle oriented upward. Upon transfer to our department, the patient presented with persistent hemiplegia, suggesting that spontaneous recovery may have plateaued and indicated a window of opportunity for active neuromodulation. Crucially, pre‐treatment diffusion tensor imaging revealed that despite the presence of a basal ganglia lesion, the macroscopic integrity of the corticospinal tract was largely preserved. This is a well‐established predictor of a favorable response to TMS therapy. Therefore, we opted for TMS and specifically selected iTBS intervention over conventional rTMS. iTBS is more efficient and can induce long‐term potentiation to enhance synaptic plasticity. We chose the cerebellar hemisphere contralateral to the lesion as the stimulation target because the patient exhibited significant reductions in balance, cognition, and mood scores upon admission. The cerebellum serves as a hub within the networks responsible for balance and emotional regulation. Compared to the Primary Motor Cortex (M1) area, it may lead to better functional improvement in patients [3]. Its anatomical location makes it easily accessible for stimulation, and it possesses neural circuits that provide a foundation for simultaneously enhancing both motor and emotional functions.

## 3 Outcome and Follow‐Up

Clinical assessments before and after treatment are summarized in Table 1. Motor function scores, including the Berg Balance Scale (BBS) and Activities‐specific Balance Confidence (ABC), showed post‐treatment improvement. Cognitive function scores, measured by the Mini‐Mental State Examination (MMSE) and Montreal Cognitive Assessment (MoCA), also improved. Additionally, anxiety and depression scores, evaluated using the Hamilton Depression Scale (HAMD) and Hamilton Anxiety Scale (HAMA), were reduced following the intervention.

**TABLE 1:** Changes in scores of patients before and after treatment.

Scale	Pre‐treatment	Post‐treatment
NIH Stroke Scale (NIHSS)	10	2
Berg Balance Scale (BBS)	51	64
The Activities‐specific Balance Confidence (ABC)	670	870
Hamilton Depression Scale (HAMD)	10	2
Hamilton Anxiety Scale (HAMA)	5	1
Mini‐Mental State Examination (MMSE)	26	29
Montreal Cognitive Assessment (MoCA)	19	26

**FIGURE 1:**
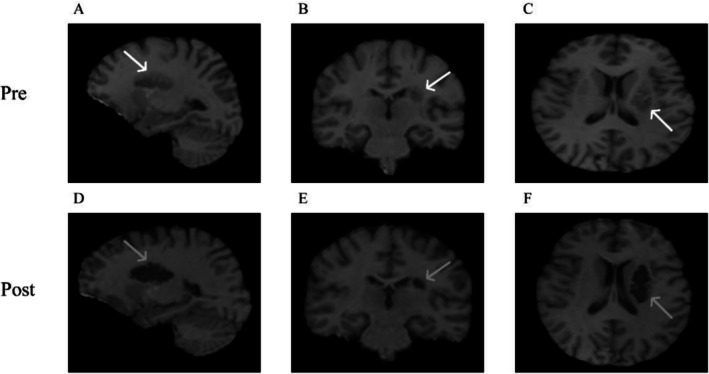
MRl of the left basal ganglia shows low signal intensity. (A–C) Before treatment; (D–F) After treatment. (White arrow: Lesion before treatment; Gray arrow: Lesion after treatment).

Functional MRI (fMRI) scans were performed before and after treatment, with results shown in Figure 1.

**FIGURE 2:**
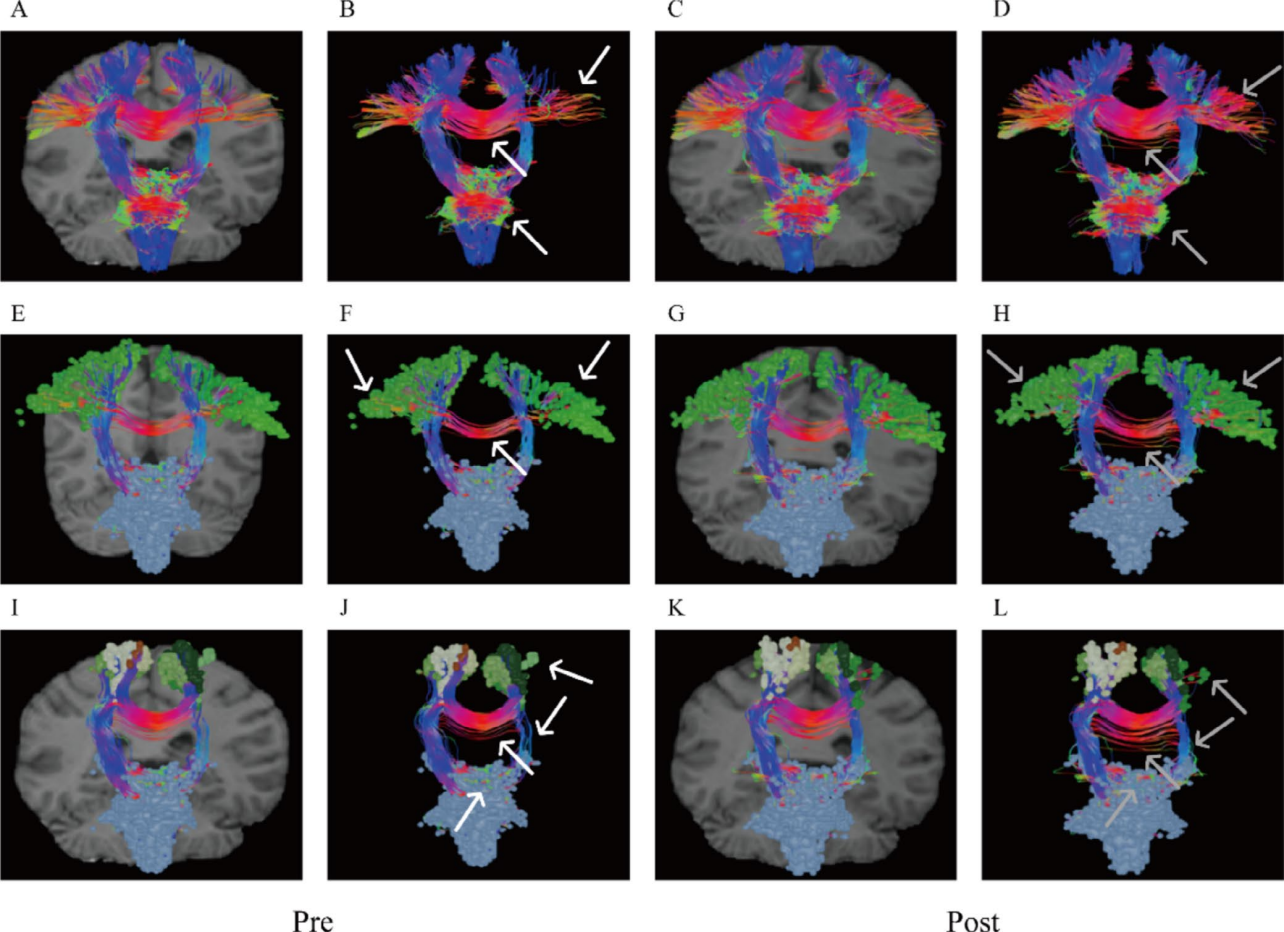
Changes in fiber connections before and after treatment. (A–D) Corticospinal tract (CST). (E–H) Primary motor cortex (M1); (I–L) Supplementary motor area (SMA). (A to B, E to F, I to J: Before treatment; C to D, G to H, K to L: After treatment; white arrows: Before treatment fiber connections; gray arrows: After treatment fiber connections).

**FIGURE 3:**
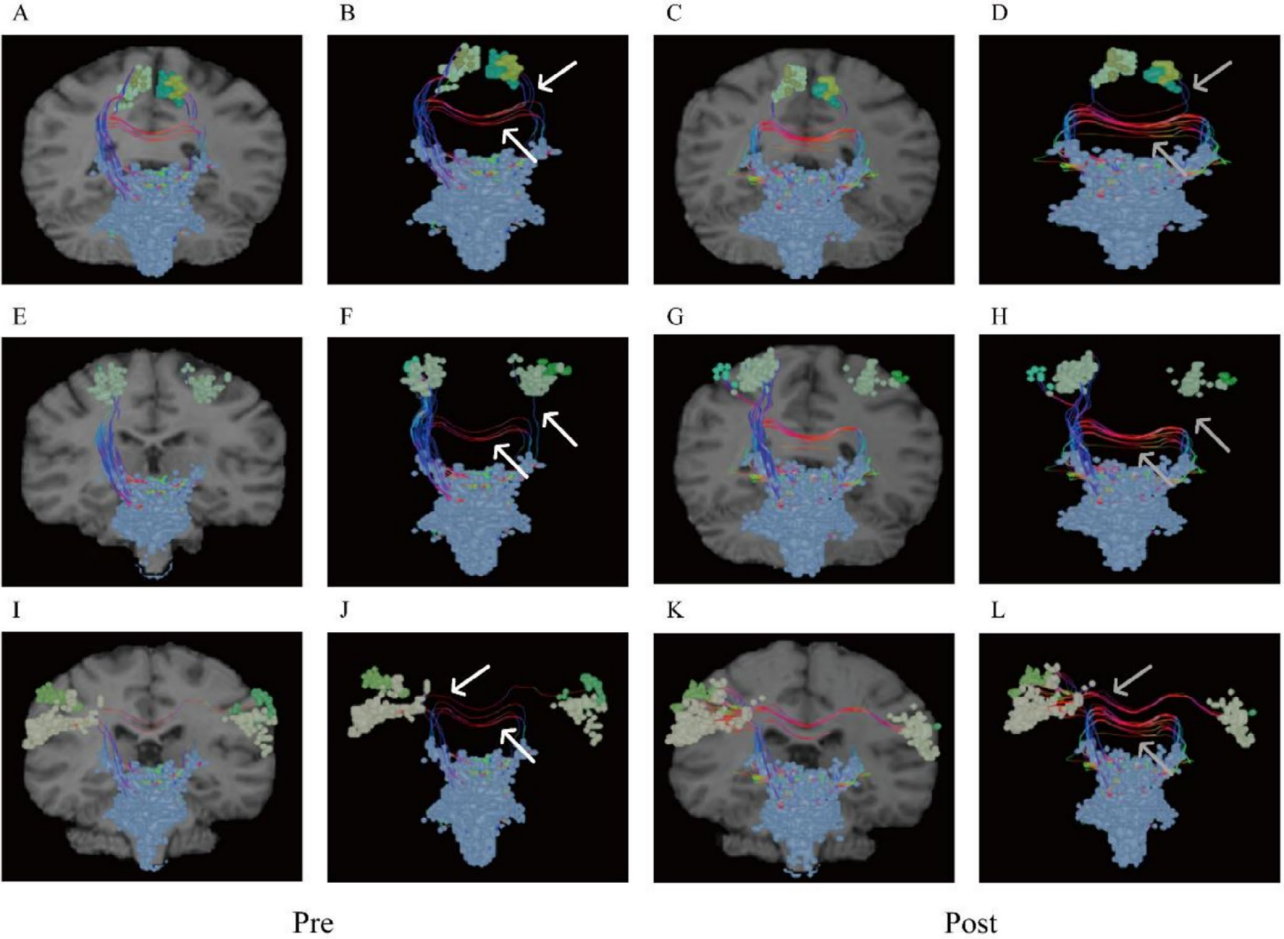
Changes in fiber connections before and after treatment. (A–D) Cingulate motor area (CMA); (E–H) Dorsal premotor cortex‐brainstem pathway; (I–J) Ventral premotor area (PMv). (A to B, E to F, I to J: Before treatment; C to D, G to H, K to L: After treatment; white arrows: Before treatment fiber connections; gray arrows: After treatment fiber connections).

Pre‐ and post‐treatment tractography and functional connectivity analyses of motor‐related fiber tracts revealed differential reactive changes across brain regions (The Human Connectome Project Multi‐Modal Parcellation (HCP‐MMP) atlas was adopted for cortical segmentation). Overall, the corticospinal tract (CST) exhibited increased connectivity in ascending and descending fibers post‐stimulation (Figure 2A–D). However, distinct patterns emerged across motor control regions: M1 (subregions 1, 2, 3a, 3b, 4; Figure 2E–H) and Supplementary Motor Area (SMA) (subregions 6ma, 6mp; Figure 2I–L) demonstrated significant increases in intra‐cortical, interhemispheric, and ascending/descending fiber connectivity; Cingulate Motor Area (CMA), Premotor Cortex‐Brainstem Pathway, and Ventral Premotor Area (PMv) showed unilateral fiber enhancement post‐iTBS, with a shift from ipsilateral to contralateral dominance relative to the lesion (Figure 3).

**FIGURE 4:**
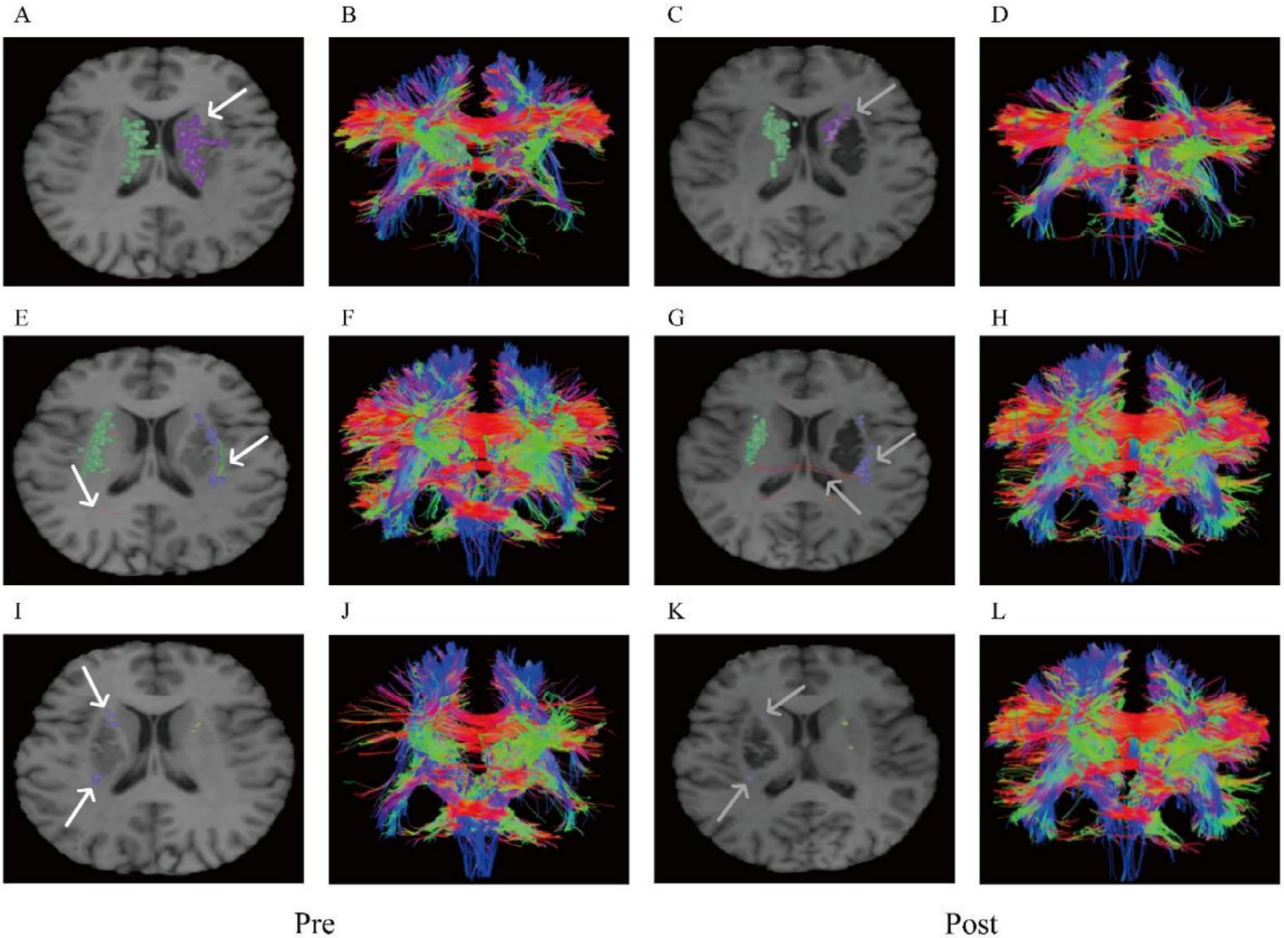
Changes in fiber connections before and after treatment. (A–D) Caudate, (E–H) Putamen; (I–J) Globus Pallidus. (A to B, E to F, I toJ: Before treatment; C to D, G to H, K to L: After treatment; white arrows: Before treatment fiber connections; gray arrows: After treatment fiber connections).

In the basal ganglia, analyses of the caudate, putamen, and globus pallidus revealed dynamic fiber reorganization during recovery. While new connections emerged from non‐lesioned to lesioned areas, the lesion core remained devoid of fiber connectivity, suggesting a compensatory growth pattern (Figure 4).

**FIGURE 5:**
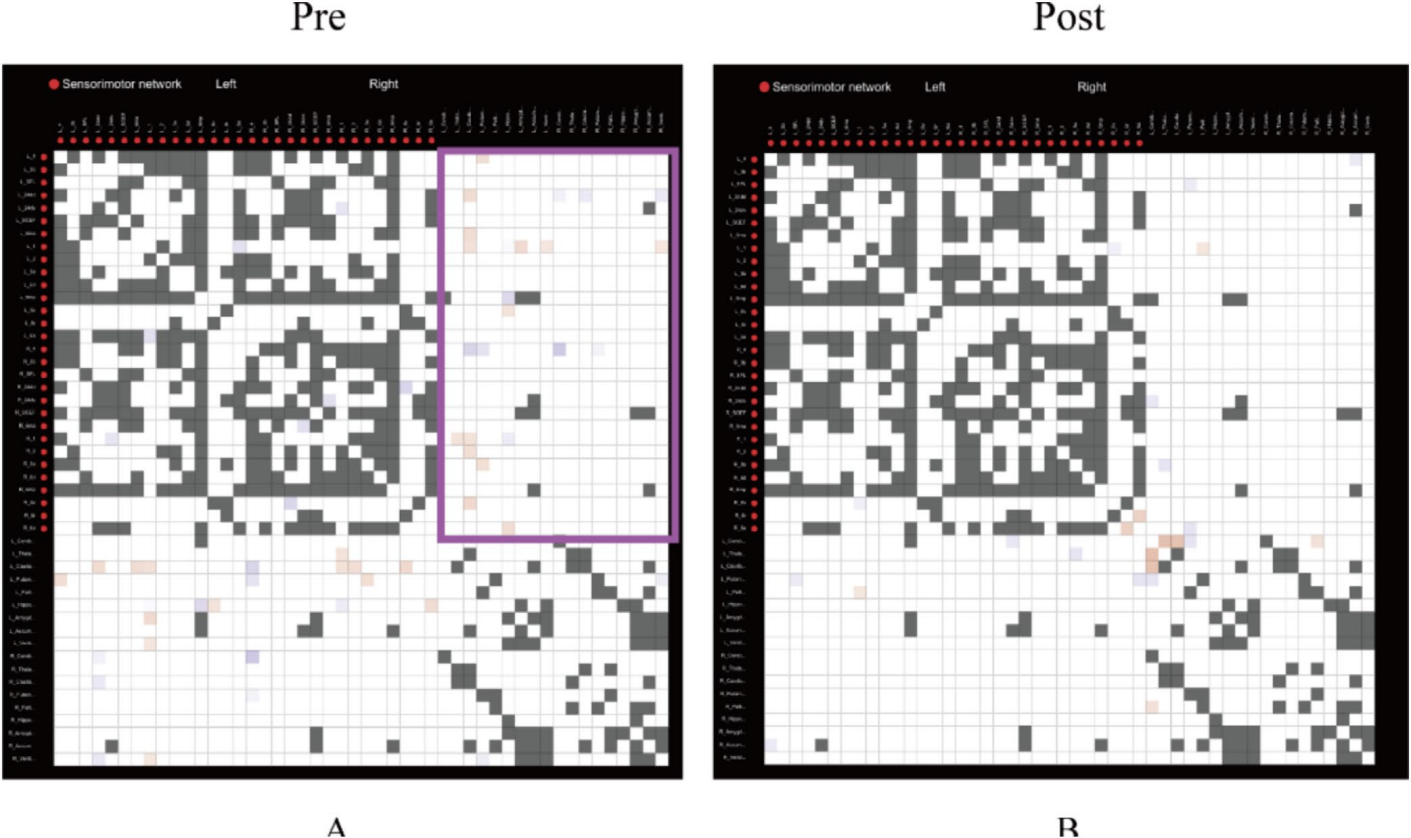
Functional connectivity matrix analysis before and after treatment. (A) Before treatment; (B) After treatment. (White: Normal; Blue: Below normal range; Red: Above normal range; Black: Natural variations can be excluded; Purple boxes: Partial abnormal connections between sensorimotor cortex and subcortical nuclei).

Regarding functional connectivity, several abnormalities were identified. Pre‐treatment (Figure 5A), in the lesioned hemisphere's sensorimotor cortex‐subcortical connections, the M1(L‐1) exhibited abnormally enhanced functional connectivity with subcortical nuclei while showing reduced connectivity within the ipsilateral cortical regions. The CMA (L‐24dd) displayed weakened connectivity with contralateral subcortical nuclei but increased connectivity with ipsilateral subcortical nuclei. Subcortical structures, including the caudate (L‐Caudate), putamen (L‐Putamen), and hippocampus (L‐HPC), demonstrated abnormally heightened cortical connectivity. In the contralateral hemisphere, M1 (R‐4) showed abnormally diminished connectivity with subcortical nuclei. Post‐treatment (Figure 5B), the most aberrant functional connections within the sensorimotor cortex were reduced; however, residual anomalies persisted: M1 (L‐1) maintained abnormal connectivity with the left pallidum (L‐Pallidum), while connectivity with the contralateral cortex (Rv6), left cerebellar subcortex (L‐Cerebellum), and left putamen (L‐Putamen) remained weakened. No complications or adverse events were observed during the follow‐up period.

## 4 Discussion

In this case report, following a 2‐week intervention of cerebellar iTBS in the patient, we observed the following: Clinically, the patient exhibited a reduction in NIHSS, HAMD, and HAMA scores after treatment, while BBS, ABC, MMSE, and MoCA scores increased, collectively indicating improvements in motor function, balance, cognition, and emotional state. At the structural connectivity level, diffusion tensor imaging revealed an overall increase in the number of fiber connections in the CST. The M1 and SMA demonstrated enhanced bilateral connectivity. Although fiber connections in the CMA, dorsal premotor area, and basal ganglia regions (caudate nucleus, putamen, globus pallidus) also increased, their orientation showed a tendency to shift toward the contralateral side, with fiber connection deficits still present in the core lesion area. At the functional connectivity level, prior to treatment, there were multiple abnormally enhanced or weakened functional connections between the sensorimotor cortex and subcortical structures. After treatment, most of these abnormal connections normalized, though some aberrant patterns persisted, such as enhanced functional connectivity between the affected primary motor cortex and the ipsilateral globus pallidus, as well as weakened connectivity with certain cortical areas on the unaffected side and the cerebellum.

Improvements in motor, cognitive, and emotional clinical scores suggest that the treatment may have produced dual efficacy by modulating the cortico‐limbic circuit, highlighting the cerebellum's dual role in motor and emotional processing [4]. The increased number of CST fiber connections likely reflects iTBS's modulatory effect on cortical excitability, thereby promoting neuroplasticity and structural reorganization [5]. The strengthened bilateral connections between the M1 and the SMA may be associated with the restoration of bilateral dominance patterns in motor control, with the enhanced connectivity in the SMA particularly facilitating the reconstruction of motor planning networks [6].

The contralateral shift in fiber connections from the CMA and dorsal premotor area may signify a transition in neural remodeling from an early bilateral activation pattern to a later contralateral compensatory mode. Like the facilitation‐inhibition mechanism in interhemispheric information integration rather than simple competition, the dorsal premotor area may compensate for functional deficits in the damaged hemisphere by enhancing connectivity [7]. Interestingly, the direction of fiber reorganization in the dorsal premotor area and the contralateral recovery of conductive fibers in the CMA suggest that the cerebello‐thalamo‐cortical circuit may mediate this process [8], with changes in connectivity in the caudate nucleus, putamen, and globus pallidus potentially following a similar pattern.

The corticobasal ganglia‐thalamocortical (CBTC) loop is widely recognized as central to motor control and feedback [9]. The “long loop” and “short loop” circuits are responsible for complex motor control/execution/feedback, respectively. Stroke patients typically exhibit weakened functional connectivity in CBTC circuits, particularly in the affected hemisphere's long and short loops [9]. Post‐stroke abnormalities in functional connectivity (both increases and decreases) suggest inhibited neuronal activity in basal ganglia regions, leading to reduced cortical inhibition and consequent cortical hyperactivation with enhanced functional connectivity [10]. This hyperactivation may represent the brain's attempt to restore lost functions through increased neural activity. Simultaneously, damaged connections between basal ganglia and thalamus impair thalamic regulation of cortical activity [9], further contributing to abnormal connectivity. TMS treatment significantly improved these connections, demonstrating its ability to enhance CBTC functional integration by modulating interhemispheric inhibition balance, promoting compensatory structural connections, and regulating cortical excitability to influence corticobasal ganglia connectivity.

In summary, the findings of this case indicate that iTBS holds potential for improving multiple functional domains in patients with basal ganglia stroke. Fiber tract and functional connectivity analyses provide visual evidence for understanding its mechanisms of action, while the distinct patterns of connectivity changes across different brain regions suggest individualized differences in neural remodeling pathways. Future large sample randomized controlled trials are still needed to validate these findings and further explore the underlying regulatory principles.

## 5 Conclusion

In conclusion, this case demonstrates TMS's therapeutic potential for post‐stroke motor dysfunction in basal ganglia hemorrhage through both clinical score improvements and neuroimaging findings. It highlights the cerebellum's dual role in motor and emotional regulation, while region‐specific recovery patterns may inform future clinical trials. However, large‐scale randomized controlled trials remain necessary to validate these findings.

## Author Contributions

**Liqing Yao:** investigation. **Lei Xi:** writing – original draft. **Qian Liu:** writing – review and editing. **Xue Yang:** investigation, writing – review and editing. **Yuzhe Zou:** visualization.

## Funding

This work was supported by: Rehabilitation Clinical Medical Centre of Yunnan Province (Grant number zx2019‐04‐02); Jiajie Expert Workstation of Yunnan Province (Grant number 2019IC034); Study on a New Model of Comprehensive Intervention in Rehabilitation and Psychology of “Brain and Heart together” (Grant number 202203AC100007‐6); Science and Technology Talent and Platform Program (Academician and Expert Workstation) (Grant number 202305AF150032); Research and Development of Integrated Chinese and Western Medicine Rehabilitation Technology and Multi‐modal Monitoring System for movement Disorders (Grant number 2022YFC2009700); National Key Research and Development Program of China (Grant number 2018YFC2002301); the Neurorehabilitation Team of Kunming Medical University (Grant number 2024XKTDTS18); Yunnan Province's Serious and Complex Diseases Combined Traditional Chinese and Western Medicine Clinical Collaboration Program—Chronic Disorders of Consciousness; Systematic Development and Industrial Applications of Balneotherapy in Stroke Rehabilitation: A Translational Research Framework (Grant number FWCY‐ZNT2024011); Application and Innovative Research of Balneotherapy in Chronic Disease Management (Grant number 202402AA310058); Scientific Research Fund project of Education Department of Yunnan Province (Grant number 2024 J0383); Doctoral research project of the Second Affiliated Hospital of Kunming Medical University (Grant number 2023BS01). Mechanism Study on Neuroplasticity in the Improvement of Post‐Stroke Depression through Intermittent Burst Stimulation of the Cerebellum (Grant number 82560452); Investigating the Impact of iTBS on the HPA Axis in Stroke Patients via Stimulation of Different Brain Regions Using Resting‐State EEG (Grant number 2025KFZD006).

## Consent

Written informed consent was obtained from the patient to publish the current case report.

## Conflicts of Interest

The authors declare no conflicts of interest.

## Data Availability

The data that support the findings of this study are available from the corresponding author upon reasonable request.
